# Out-of-home participation among people living with dementia: A study in four countries

**DOI:** 10.1177/14713012221084173

**Published:** 2022-04-17

**Authors:** Liv Thalén, Camilla Malinowsky, Isabel Margot-Cattin, Sophie N Gaber, Kishore Seetharaman, Habib Chaudhury, Malcolm Cutchin, Sarah Wallcook, Kottorp Anders, Anna Brorsson, Louise Nygård

**Affiliations:** Division of Occupational Therapy, Department of Neurobiology, Care Sciences and Society, 27106 Karolinska Institutet, Huddinge, Sweden; Division of Occupational Therapy, Department of Neurobiology, Care Sciences and Society, 27106 Karolinska Institutet, Huddinge, Sweden; Department of Occupational Therapy, School of Social Work and Health, Lausanne (HETSL), University of Applied Sciences and Arts of Western Switzerland (HES-SO), Delémont, Switzerland; Division of Occupational Therapy, Department of Neurobiology, Care Sciences and Society, Karolinska Institutet, Ersta Sköndal Bräcke University College, Department of Health Care Sciences, and Uppsala University, Department of Women’s and Children’s Health, Clinical Psychology in Healthcare, Huddinge, Sweden; Department of Gerontology, 1763 Simon Fraser University, Vancouver, BC, Canada; 144477 Pacific Northwest University of Health Sciences, Yakima, WA, United States; Division of Occupational Therapy, Department of Neurobiology, Care Sciences and Society, 27106 Karolinska Institutet, Huddinge, Sweden; Division of Occupational Therapy, Department of Neurobiology, Care Sciences and Society, Karolinska Institutet, Sweden and Faculty of Health and Society, Malmö University, Malmö, Sweden; Division of Occupational Therapy, Department of Neurobiology, Care Sciences and Society, 27106 Karolinska Institutet, Huddinge, Sweden

**Keywords:** society, community, environment, neighbourhood, place

## Abstract

Social participation in out-of-home activities is important for people living with dementia, yet little is known about such participation. The aim of this study was to explore and compare out-of-home participation among people living with dementia in four countries by assessing different types of places of participation visited or no longer visited. A cross-sectional design was used to gather self-reported experiences concerning out-of-home participation among people with mild stage dementia living in Canada (*n* = 29), Sweden (*n* = 35), Switzerland (*n* = 35) and the UK (*n* = 64). Interviews were conducted using the *Participation in ACTivities and places OUTside the Home for older adults* (ACT-OUT) instrument. Participants still visited 16 (*Median*) places out of a possible total of 24, and they had abandoned 5 (*Median*) places. Neighbourhood was the place most participants still visited, whereas 50% of them had stopped going to a Sports facility, with no significant differences between country samples regarding how many participants had abandoned that place (Fisher’s exact test, *p* > 0.01). There were significant differences between country samples in the frequency of present participation and abandonment of the Hospital, Dentist’s office, Cemetery, Garden, and Forest (Fisher’s exact test, all *p* < 0.01). Although the participants still visited a variety of places, they had stopped going to places previously visited, which indicates reductions in participation, posing an inherent risk to well-being. The similarities and differences across samples from the four countries suggest that healthcare services and access to public transport may contribute to the complex interactional process of out-of-home participation for people living with dementia. The findings highlight the need for initiatives targeting specific types of places to support continued participation in society, especially places at a higher risk of abandonment such as places for recreation and physical activity.

## Background and objectives

The Convention on the Rights of Persons with Disabilities (United Nations, 2006) states that regardless of disability, every person has the right to full participation and inclusion in society, including but not limited to participation in political and public life (article 29) and cultural life, recreation, leisure and sport (article 30). Participation has been broadly defined in the International Classification of Functioning, Disability, and Health (ICF) as one’s involvement in a life situation, for example, community, social, and civic life (World Health Organization, 2001). Nowadays, with more people being diagnosed with dementia in an earlier stage (Dubois et al. (2016), many individuals with dementia are living at home. However, research has shown that going out of the home to different places for different activities may be challenging for people living with dementia (Brorsson et al., 2011, 2016; Brorsson et al., 2013), which also affects their participation (Margot-Cattin et al., 2021; Gaber et al., 2020a). To date, the question of how people with mild and moderate dementia reduce participation in society by decreasing their visits to places where affairs of life are conducted outside the home has not been investigated.

Participation in activities and places outside home is known to be an important contributor to the social health of people living with dementia (Sturge et al., 2020). Through the maintenance of out-of-home activity and participation, people living with dementia are known to experience normality and continuity (Chen et al., 2019) as well as autonomy, mastery, freedom, and enhanced well-being (Odzakovic et al., 2018; Phinney et al., 2016). Moreover, participation in out-of-home activities can be a valuable source of emotional, practical and social support for people living with dementia (Clark et al., 2020). Conversely, the degree of social, emotional or practical support and other psychosocial benefits that one derives from participating in the community could help motivate and shape out-of-home activity and participation for people living with dementia (Li et al., 2019; Odzakovic et al., 2018; Ward et al., 2018).

Among people living with dementia, more preserved cognitive function may correlate to a wider range of activities (Chiu et al., 2013). People with dementia may participate less in more complex and cognitively demanding tasks performed out of home, such as volunteering, conducting business at the bank or visiting the library, compared to people without dementia (Wahl et al., 2015). Previous research also shows that people living with dementia have progressively smaller spatial ranges of out-of-home activity over time (Shoval et al., 2011). Hence, out-of-home activity tends to occur in locations closer to one’s home (Duggan et al., 2008; Shoval et al., 2011), and consist of routinised activities (Wahl et al., 2015). Taken together, previous research has indicated that a focus on visits to and use of (maintained or abandoned) places in community, and the activities that typically are performed in these places, might provide a more nuanced picture of participation among older adults with and without dementia. This led us to develop the *Participation in ACTivities and places OUTside the Home for older adults* (ACT-OUT) survey tool, and this was simultaneously done in three languages (English, French and Swedish) using a pre-defined method to foster internal validity (Margot-Cattin et al., 2019; Margot-Cattin, 2021). Previous studies have utilised and contributed to the validation of the ACT-OUT; where patterns of community participation among older adults with and without dementia have been investigated (Chaudhury et al., 2021; Gaber et al., 2020a; Gaber et al., 2019; Gaber et al., 2020b; Margot-Cattin et al., 2021; Wallcook et al., 2020), and among people diagnosed with stroke (Malinowsky et al., 2019; Olofsson et al., 2019).

Modifications and adaptations to built, social and supportive service environmental factors can have a wide-ranging effect on out-of-home participation for persons living with dementia and contribute to create Dementia-Friendly Communities (Gaber, 2021). Besides the neighbourhood built environment, other aspects, such as geographical, demographic, social and organisational aspects, city planning and healthcare authorities may also contribute to how a person experiences out-of-home participation in different activities and places (Gaber, 2021; Margot-Cattin, 2021). This operationalisation of out-of-home participation in the ACT-OUT survey allows systematic examination of the topic, thereby shedding light on commonalities as well as differences among environments. To our knowledge, there is no previous research that explores and compares patterns of out-of-home activity and participation in common places of social participation in samples from multiple countries. The aim of this study is to explore and compare out-of-home participation patterns among people living with dementia in four countries. The analyses include present places of participation patterns as well as patterns of change in places of participation from past to present. A secondary aim is to compare patterns across the four country samples.

## Research design and methods

A prospective, cross-sectional design was used to explore and gather data on the complex area of participation outside home among people living with dementia in Canada, Sweden, Switzerland and the United Kingdom. An overview of methodological aspects, including the types of settings where participant recruitment took place, is presented in Table 1. These four countries have several similarities: a relatively large proportion of people aged 65 or more (17–20%), high income standings, and similar initiatives on the promotion and implementation of dementia-friendly societies (see, for example, the Canadian National Dementia Strategy (Public Health Agency of Canada, 2019), the Swedish National Guidelines on Dementia (National Board of Health and Welfare, 2010), the Swiss National Alzheimer Plan [Office Fédéral de la Santé Publique, 2016) and the UK Dementia strategy (Department of Health, 2009)]. On the other hand, differences are found across the countries within areas such as geographical settings, organisation of health care, public transportation and subsidies for travels. The inclusion of these particular four countries occurred organically based on the strength of research networks. Individual studies were initially conducted in each country; hence, the samples were not matched. Nonetheless, the resulting combined sample provides an opportunity to closely examine stability and change in participation in places outside home. As recommended by Ward et al. (2021), including data from multiple countries allows location to be taken into account in investigation of participation, rather than merely focusing on disease symptoms. Although contextual socio-environmental data in the four countries were not explicitly collected, the similarities and differences of out-of-home participation among people with dementia in the four countries would provide valuable insights, and generate research questions and relevant methods for future investigations. This paper has been co-developed by authors from the four countries with a collaborative approach; all authors have provided valuable insights and participated in discussions throughout all phases of the study and writing of this paper.

**Table 1.** Demographic characteristics of participants by country.

		Country				
		Canada	Sweden	Switzerland	United Kingdom	Differences between country
Setting		Vancouver: urban and suburban area	Stockholm region: urban and suburban areas	French speaking regions: Small cities, rural areas, mountains	London, Cumbria, Greater Manchester regions: urban and rural areas	N/A
Recruited through		Dementia screening centre	Memory clinics and community-based groups	Memory clinics, day hospitals and Swiss’ Alzheimer’s association	National Health Services centres and community-based activities	N/A
Variable	Category	Count (% within country)				
Gender	Male	**15** (54%)	**13** (37%)	**16** (46%)	**35** (55%)	*n.s*.^a^
	Female	**13** (46%)	**22** (63%)	**19** (54%)	**29** (45%)	
Age	≤64 years	**6** (21%)	**2** (6%)	**3** (9%)	**2** (3%)	*n.s*.^b^
	65–74 years	**12** (41%)	**15** (43%)	**10** (29%)	**15** (23%)	
	75–84 years	**9** (31%)	**14** (40%)	**14** (40%)	**34** (53%)	
	≥85 years	**2** (7%)	**4** (11%)	**8** (23%)	**13** (20%)	
Level of education	Primary and/or Secondary School	**1** (3%)	**13** (37%)	**4** (11%)	**16** (25%)	*p* < 0.01^b^
	High school/GED/apprenticeship	**9** (31%)	**11** (31%)	**22** (63%)	**38** (60%)	
	Degree from University/College	**19** (67%)	**11** (31%)	**9** (26%)	**10** (16%)	
Living condition	Alone	**4** (14%)	**19** (54%)	**11** (31%)	**25** (39%)	*p* < 0.01^b^
	Co-habiting	**24** (86%)	**16** (46%)	**24** (69%)	**39** (61%)	
Supporting person available	No	**1** (4%)	**1** (3%)	**4** (11%)	**3** (5%)	*n.s*.^b^
	Yes	**27** (96%)	**31** (97%)	**31** (89%)	**61** (95%)	
Driving car oneself	No	**23** (82%)	**31** (89%)	**28** (80%)	**38** (59%)	*p* < 0.01^b^
	Yes	**5** (18%)	**4** (11%)	**7** (20%)	**26** (41%)	
Transportation Service	No	**18** (67%)	**9** (26%)	**31** (89%)	N/A	*p* < 0.01^b^
	Yes	**9** (33%)	**26** (74%)	**4** (11%)	N/A	
MOCA	Median score (0–30)	N/A^d^	**19** (10–30)	**18** (8–29)	**21** (12–28)	*n.s*.^c^

*Note*. MOCA = Montreal cognitive assessment.

^a^Analysed with Pearson’s χ^2^.

^b^Analysed with Fisher’s exact test^.^

^c^Analysed with Kruskal–Wallis’ H test^.^

^d^The recruiting health centre had already screened the study participants ensuring they were in the mild-moderate stages of dementia, that is, MMSE scores equal or more than 10. The actual MMSE scores of some of the participants were not disclosed by the centre due to privacy restrictions. Hence some participants’ scores were unavailable. (Folstein et al., 1975)

### Participants

In total, 163 participants were included from Canada (*n* = 29), Sweden (*n* = 35), Switzerland (*n* = 35) and the United Kingdom (UK, *n* = 64). Shared inclusion criteria were to have a diagnosis of mild to moderate stage dementia and to live at home. The one exclusion criterion was the inability to communicate during an interview, for example, due to language barriers or severe hearing or vision impairment that was not compensated by aids. The participants were recruited through a dementia screening centre (Canada), memory clinics (Sweden, Switzerland), Swiss Alzheimer’s association (Switzerland), National Health Service centres (the United Kingdom). Complementary information considering participants in each country is found in Table 1. The recruitment procedures in each country have been described in more detail elsewhere (Chaudhury et al., 2021; Gaber et al., 2019; Gaber et al., 2020b; Margot-Cattin et al., 2021). All interviews were undertaken within the same time frame 2015–2017, and all interviewers were experienced in interviewing people living with dementia, and trained in using the ACT-OUT. In Sweden, all interviews were done by three specifically trained researchers, in Switzerland by two, in the UK by two, and in Canada by one. Participants were invited to have a significant other present as support in the interview situation if they wished, but the person with dementia was always the responding person.

No significant differences were found between the country samples based on gender, age and whether participants had someone who could help them. Significant differences were found for the variables level of education, living alone or together with someone, driving a car or not, and if they had been granted a public transportation service or not (all *p* < 0.01). However, there were only two significant post hoc tests; Sweden had a significantly higher rate of granted public transportation service than expected, whereas Switzerland had a significantly lower rate (both *p* < 0.001 with Bonferroni correction).

Each participant chose the location for the interview and was offered to bring an accompanying person.

### Material

Data were collected using the *Participation in ACTivities and places OUTside the Home for older adults* (ACT-OUT) survey tool designed to examine the out-of-home participation of older adults (with or without dementia) in relation to places over time. In the development of the ACT-OUT, questions were trialed with older adults without as well as with dementia (Margot-Cattin et al., 2019). The ACT-OUT questionnaire consists of three parts, where Part 1 is divided into four domains: A) Consumer, administrative and self-care places (*n*= 6), B) Places for medical care (*n* = 5), C) Social, cultural and spiritual places (*n* = 6) and D) Places for recreational and physical activities (*n* = 7). Part 1 consists of yes/no questions regarding present, previous, and anticipated future participation in activities at the places listed. In Part 2, more detailed questions are asked about a couple of places in each domain, for example, related to activities undertaken in the place, frequency of visits, distances and modes of transport. In Part 3, questions about attitudes to risk-taking are asked. In this study, only data from Part 1 were used because our aim was to investigate participation in places at present and in the past across the four countries. In order to harmonise the data collection, all interviewers at each site followed the same procedures by paying close attention to the user manual of the ACT-OUT and they could raise any questions to the research group who constructed the ACT-OUT (Margot-Cattin et al., 2019). The length of interviews varied between 45 and 120 min, as we tried to be responsive to the needs of each participant and adapted the duration accordingly, that is, if breaks were required.

### Data analysis

Similarities across countries were descriptively narrated. Differences between the participants from the four countries were significance tested with non-parametric statistical methods since data either were nominal or ordinal that did not show the same shape of distribution for each group. Depending on the level of data, number of groups, and assumptions fulfilled, analyses were made either with Kruskal–Wallis H test (with *r* calculated for effect size and Mann–Whitney *U* test used for post hoc analyses), Mann–Whitney *U* test (with *r* calculated for effect size) or Fisher’s exact test (with adjusted residual/probability values used for post hoc analyses). The significance value was set conservatively, *p < 0.01, to reduce the risk of Type 1 errors, and Bonferroni corrections proceeded from that.

### Ethical considerations

Information about the study was given verbally as well as in writing before informed consent was obtained. Each participant’s capacity to give informed consent was carefully assessed by the experienced interviewers before the interviews. Measures were taken to ensure that participants felt safe during the interviews. All participants were informed that their participation was voluntary and that they could opt out at any time, without any explanation. Interviewers were alert to notice any indication of a participant getting uncomfortable or tired. Ethical approvals were granted by the *Office of Research Ethics at Simon Fraser University* (2017s0052) for the Canadian sample, the *Regional Board of Research Ethics* (2015/77-31-5) for the Swedish sample, the *Commission cantonale d'éthique de la recherche sur l'être humain in Lausanne* (protocol 452/15) for the Swiss sample and the *Health Research Authority* (IRAS project ID: 215654, REC reference: 17/SW/0091) for the sample from the UK.

## Results

### Places visited outside home

Of the 24 places outside home included in the ACT-OUT, the median number of places all participants (*N* = 163) visited was 16 (0–22). In Table 2, the median number of places visited and abandoned for each domain is presented by country. A Kruskal–Wallis H test showed a statistically significant difference between countries for the median number of visited places, *χ*^*2*^ (3) = 19.03, *p* < 0.01, with a mean rank of 107.57 for participants from Sweden, 80.81 for UK, 79.71 for Switzerland and 56.52 for Canada. Pairwise comparisons showed no significant differences, except for a significantly higher number of places visited in the Swedish sample (*Mdn* = 18) compared to the Canadian (*Mdn* = 13), *U* = 209.50, *z* = −4.04, *p* < 0.001, *r* = −0.50. The effect measure r indicates a large effect size accounting for 25% of the variability.

**Table 2.** The median number of places outside home in the ACT-OUT visited and abandoned by country: total number and per Domain.

	Canada (*n* = 29)	Sweden (*n* = 35)	Switzerland (*n* = 35)	UK (*n* = 64)	Kruskal–Wallis H tests’ sign. values (*p*)	Countries where post hoc analysis (Mann–Whitney U tests) showed sign. diff. (*p* < 0.001)
Total number of places visited, **Mdn** (Min–Max)	13.00 (0–20)	18.00 (2–21)	15.00 (8–21)	16.00 (8–22)	*p* < 0.01	More places visited in Sweden compared to Canada
Domain A^a^, visited	4.00 (0–6)	5.00 (0–6)	5.00 (0–6)	5.00 (0–6)	*n.s.*	—
Domain B^b^, visited	2.00 (0–4)	3.00 (2–5)	3.00 (2–5)	3.00 (1–4)	*p* < 0.01	More places visited in: Sweden compared to Canada; Switzerland compared to Canada
Domain C^c^, visited	3.00 (0–6)	5.00 (0–6)	4.00 (1–6)	3.00 (1–6)	*p* < 0.01	More places visited in: Sweden compared to Canada; Sweden compared to the UK
Domain D^d^, visited	4.00 (0–6)	5.00 (0–6)	4.00 (1–7)	5.00 (0–7)	*n.s.*	—
Total number of places abandoned, **Mdn** (Min–Max)	6.50 (2–14)	3.00 (0–18)	5.00 (1–11)	4.50 (0–11)	*n.s.*	—
Domain A, abandoned	1.00 (0–5)	0.00 (0–6)	1.00 (0–6)	0.00 (0–5)	*n.s.*	—
Domain B, abandoned	1.00 (0–3)	0.00 (0–3)	1.00 (0–2)	1.00 (0–3)	*p* < 0.01	More places abandoned in the UK compared to Sweden
Domain C, abandoned	2.00 (0–5)	0.00 (0–6)	1.00 (0–3)	1.00 (0–4)	*p* < 0.01	More places abandoned in Canada compared to Sweden
Domain D, abandoned	2.00 (0–5)	2.00 (0–7)	1.00 (0–5)	1.00 (0–5)	*n.s.*	—

*Note*. Domain A = Consumer, administration, and self-care places, Domain B = Places for medical care, Domain C = Social, spiritual, and cultural places, Domain D = Places for recreation and physical activities.

**Figure 1.**
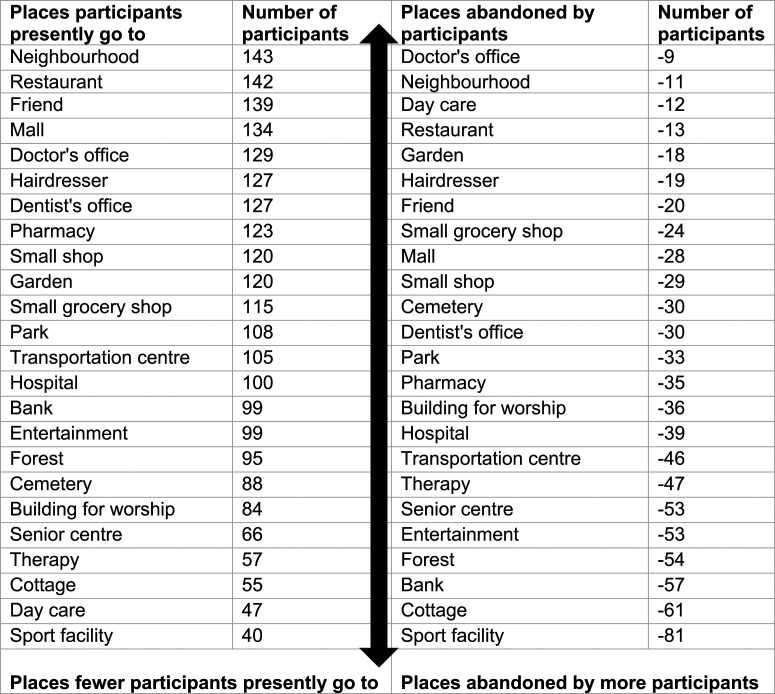
Number of participants (from all countries) who presently visited/abandoned places in ACT-OUT.

Focusing on the listed places within the ACT-OUT, Figure 1 shows the total number of participants (combining all four samples) who visited them.

**Figure 2.**
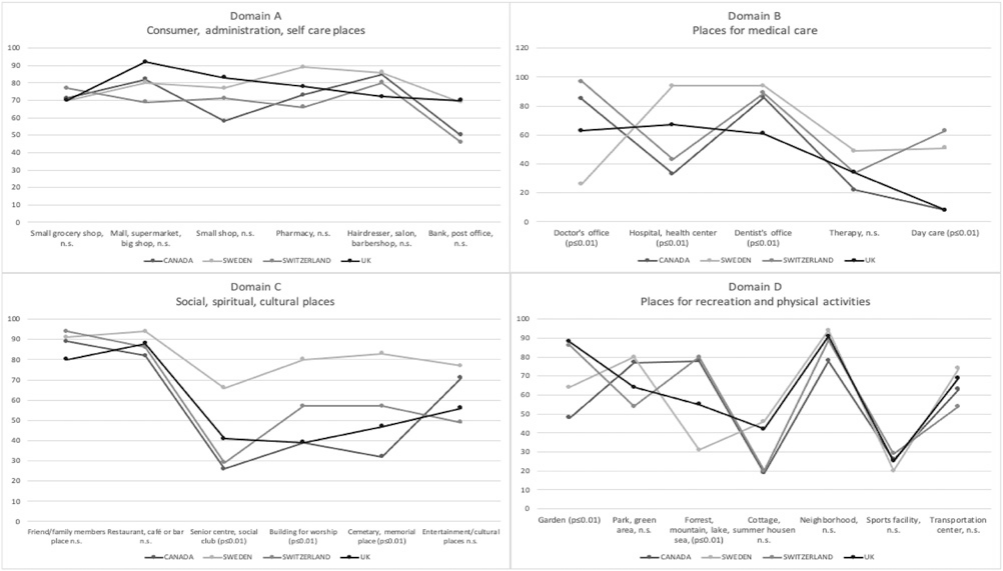
Percentages of participants within country who visited the places in ACT-OUT. Fisher’s exact test has been used to investigate any significant differences between countries, *p* < 0.01.

Figure 2 shows the percentages of participants who visited each place by country in four graphs, one for each domain. Fisher’s exact tests were utilised to investigate any statistically significant differences regarding the number of participants who visited each place by country. Significant differences (*p* < 0.01) were found between samples from the different countries for the places Doctor’s office, Hospital/Health centre (hereinafter named Hospital), Dentist’s office, Day care, Senior centre/Social club (hereinafter named Senior centre), Building for worship, Cemetery/Memorial place (hereinafter named Cemetery), Garden and Forest/Mountain/Lake/Seaside (hereinafter named Forest). Post hoc tests were performed with adjusted residual values for the places where Fisher’s exact test had shown an overall statistically significant difference between samples from different countries. Post hoc analysis showed *no* statistically significant differences, with Bonferroni correction, for the places Day care, Senior centre and Building for worship, all *p* > 0.001. Only probability values that differed significantly from the null hypothesis with Bonferroni correction (*p* < 0.001) are presented for the places Doctor’s office, Hospital, Dentist’s office, Cemetery, Garden and Forest: Within country, the Swedish sample had statistically significantly fewer participants than expected, 26%, going to the Doctor’s office. In the UK-sample on the other hand, there was a significantly higher rate than expected, with 98% of participants going to the Doctor’s office. The Swedish sample had a significantly higher rate (94%) of participants going to Hospital. The UK-sample had a significantly lower rate (61%) of participants going to the Dentist’s office. The Swedish sample had a significantly higher rate (83%) of participants going to the Cemetery, whereas the Canadian sample had a significantly lower rate (32%). The UK-sample had a significantly higher rate (88%) of participants going to the Garden, whereas the Canadian had a significantly lower rate (48%). Finally, the Swedish sample had a significantly lower rate (31%) of participants who were going to Forest.

### Abandoned places outside home

The median number of places the participants had stopped going to were five (min -max: 0–18). A Kruskal–Wallis H test showed no statistically significant difference between samples from different countries, *χ*^*2*^ (3) = 6.81, *n.s*, with a mean rank of 91.94 for participants from Canada, 78.94 from Switzerland, 75.34 from the UK and 60.41 from Sweden.

**Figure 3.**
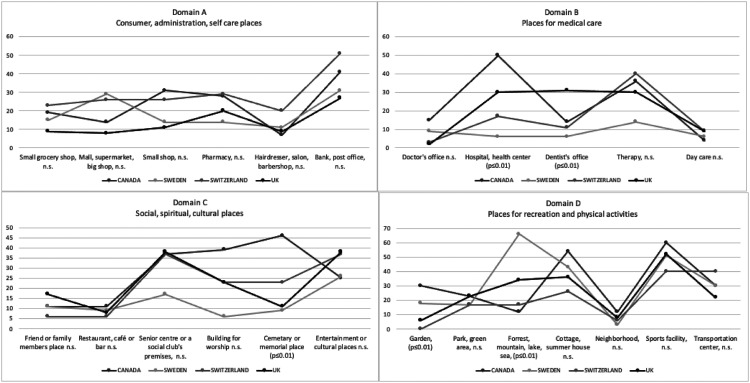
Percentages of participants within countries who had abandoned the places in ACT-OUT. Fisher’s exact test has been used to investigate any significant differences between countries, *p* < 0.01.

Looking into each place, Fisher’s exact tests were utilised to investigate any statistically significant differences regarding the number of participants who had abandoned each place by country sample. Significant differences (*p* < 0.01) were found for the Hospital, Dentist’s office, Cemetery, Garden and Forest. Figure 3 shows the percentages of participants within country sample who had abandoned each place in four graphs; one for each Domain A-D. Post hoc tests were performed with adjusted residual values for the five places where a Fisher’s exact test had shown an overall statistically significant difference between country samples. For the Hospital, Dentist’s office, Cemetery and Garden, post hoc analyses showed no statistically significant differences, all *p* > 0.001. The Swedish sample had a significantly higher rate of participants than expected (31%) who had stopped going to Forest, *p* ≤ 0.001. No other countries’ probability values differed significantly from the null hypothesis. All five places where there was a significant difference between countries with regard to the rate of abandonment *also* had a significant difference between countries with regard to present participation in that place.

## Discussion and implications

The aim of this study was to explore and compare different aspects of stability and change in visits to places outside home among people living with mild to moderate dementia using samples recruited from four countries.

Participants in all countries visited places outside home, and there was a significant difference among participants across countries in the median number of places visited. Specifically, participants from Sweden visited significantly more places than participants from Canada. Assuming the samples were similar enough to be compared with an intention to learn more about participation, differences such as these are useful for reflection and future inquiry. Canada and Sweden have a comparably large land area per capita, but most citizens live in urban or suburban areas including the samples in these two countries. However, there are differences in city planning and urban morphology between the countries, where cities in Sweden tend to have higher residential density and designated sidewalks, compared to Canadian cities. Previous research suggests that higher residential density contributes to higher concentration of services or amenities; a neighbourhood with access to more service facilities may provide easier access to these places, and familiarity of neighbourhood may facilitate continued out-of-home participation (Olsson et al., 2019; Margot-Cattin et al., 2021; Sandberg et al., 2015; Wahl et al., 2015). Additionally, differences in the sample group characteristics in the four countries may influence the levels of participation. A few variables, such as level of education, living alone or with someone, driving a car, were significantly different across the countries. More research is needed to explore the role of the contextual socio-environmental and demographic factors on continued participation, since only two of the post hoc tests were found statistically significant.

Over 80% of all participants visited a Neighbourhood (Domain D), Restaurant (Domain C), Friend/family member’s place (from here named Friend’s place, Domain C), Mall (Domain A) and Doctor’s office (Domain B). No statistically significant differences were found across countries for any of these five places. Participants visiting these five places is consistent with previous research which has indicated the importance of going to the neighbourhood and shopping (Brorsson et al., 2011, 2013, 2016; Odzakovic et al., 2018), and also an increase in visits to health-related places (Chaudhury et al., 2021) for people living with dementia. However, for 7/24 places listed in the ACT-OUT, there was a significant difference across samples from the four countries regarding present participation, with most of them found in Domain B (Doctor’s office, Hospital, Dentist’s office, Day care) and C (Senior centre, Building for worship, Cemetery). On the other hand, only two places in Domain D (Garden, Forest) and zero places in Domain A showed a statistically significant difference between samples from different countries. One explanation could be that the places in Domain A may be more closely connected to so called essential out-of-home activities, upon which the participants depend for their daily living (for example grocery shopping). Interestingly, earlier research among people living with dementia has shown that places such as the grocery store also might be experienced as places for social interaction and going there as an opportunity to physical exercise (Brorsson et al., 2011), suggesting that places might take on multiple meanings. Another explanation for the differences in the number of visits to a Doctor’s office, Hospital, Dentist’s office, and Day care in different countries could be related to variation in health care services and financial subsidies from government.

Although the participants still visited several places, they had stopped going to places previously visited. No significant difference was found between the samples from different countries regarding the overall rate of abandonment. Looking into each domain, the same pattern emerged as for the presently visited places; a statistically significant difference was only found for Domain B and C. Participants from the Swedish sample had abandoned significantly fewer places for medical care (Domain B) compared to the UK-sample, with a medium effect size. For social, spiritual and cultural places (Domain C), participants from Sweden had abandoned fewer places than participants from Canada, with a medium effect size. A pattern emerges, suggesting that people with dementia living in Sweden may experience facilitators to access various places. Another explanation may be sought within transportation and the journey to and from a place.

A specific barrier for people living with dementia is the eventual loss of driving privileges. This can increase dependence on others for transportations to destinations or alternative modes of transport. The consequence is decreased participation in non-essential out-of-home activities compared to those that are considered more essential, for example, medical appointments, or household, for example, grocery shopping (Taylor & Tripodes, 2001). The Stockholm area has extensive public transportation possibilities, like commuter trains, metro, tram and buses. Many of these means of travel have adaptations to allow people with mobility difficulties to travel by public transport. Beyond physical access, people with dementia can also apply for a transportation service, which, if granted, gives free passage on all public transports. Support to travel, such as a concession travel pass (Musselwhite, 2018), has earlier been found to contribute to overall participation outside home among people living with dementia in UK (Gaber et al., 2020a). More in-depth research is needed to explore accessibility in terms of modes of transportation, neighbourhood built environmental features, and public transit options.

A wide range was noted in the number of participants who had abandoned a place: less than 1% had stopped going to a Doctor’s office, but 50% had stopped going to a Sports facility. The places fewest people had abandoned were a Doctor’s office (Domain B), Neighbourhood (Domain D), Day care (Domain B), Restaurant (Domain C) and Garden (Domain D). The places Doctor’s office, Neighbourhood and Restaurant were also among the top five most frequently visited places presently. The coincidence of high participation in the present time and low rate of abandonment may indicate that several people living with dementia across countries want to, are able to, and have access to visit these places.

On the other hand, places with the largest rate of abandonment were Sports facility (Domain D), Cottage (Domain D), Bank (Domain A), Forest (Domain D). The places Sports facility and Cottage were also found at the bottom of the hierarchy of present participation. The difference between previous and present participation was large; only 40 participants went to a Sports facility presently, but 81 did go there before. The large rate of abandonment indicated that either the participants once had more interest in going there, or that they had lost access to these places. Previous data from Canada highlighted that places more frequently abandoned by people living with dementia should be examined closely when striving to make communities more dementia-friendly (Chaudhury et al., 2021). Our findings add to this and send an important message to stakeholders to specifically address older peoples’ access to nature places and sports facilities as part of developing dementia-friendly and inclusive communities (ADI, 2017). This is aligned with the Convention on the Rights of Persons with Disabilities (CRDP) that states that all people, including those living with dementia, have the right to live independently and participate fully in all aspects of life (United Nations, 2006).

### Limitations and future research

Importantly, we do not claim to generalise the findings to the countries where data have been collected. Rather, our ambition is to shed light on the topic of participation in out-of-home places among people living with dementia across a variety of environments that are both similar and different. A cross-national perspective and understanding of destinations in people’s everyday routine seem timely and relevant given the global emphasis on creating dementia-friendly communities and enhancing community participation among people living with dementia. Yet, the samples are small and unmatched, and for several demographic aspects, the groups of participants differ significantly from each other across the countries. This discrepancy includes two important variables: transportation, namely, if participants are driving a car themselves, and whether they use a public transportation service. It has earlier been pointed out that driving cessation following dementia usually has consequences for the persons’ participation in places outside home (Ward et al., 2021). The availability of transportation options to different places in the community and location of services would therefore be of interest to explore in a future study. There might be variations in visits outside the home for people living in rural areas who use an automobile as their only means of transportation and those who live in urban areas with access to public transportation.

We need to also acknowledge that there are weaknesses in the ACT-OUT tool that might have influenced the findings. For example, when we ask about the past, we did not define the timeframe for ‘past’, hence there might be variations in how ‘past’ was understood by participants. In addition, we did not ask about frequency of visits to places in the past versus the present, so such changes might have escaped under our radar (e.g. a person might still occasionally visit a place, but much less frequently than earlier). Furthermore, there are variations in access to healthcare services across the four countries in terms of the location of services in the community and the affordability of the services depending on private or public healthcare systems. Socio-environmental or external factors, such as social deprivation of the living environment, may be of more importance in influencing the number of places a person goes to, compared to the influence of individual or internal factors (Wallcook et al., 2020). Future research could examine contextual factors, such as rural versus urban areas, healthcare systems, socio-economic factors and built environmental, and the possible effects on community engagement and use of health care and other services. Qualitative research methods could contribute to more in-depth understanding of the reasons and motivations behind participants maintaining and abandoning certain places.

## Conclusion

The study showed that participants from samples in Canada, Sweden, Switzerland and the UK visited a variety of places outside home, despite also having abandoned several places. To support quality of life for people living with dementia through independent and safe mobility and access to places in nature and in the community, it is important to identify places that tend to be abandoned, such as sports facilities, where there is a need to take measures to facilitate continued participation in out-of-home activities. In this endeavour, the views of people living with dementia should be a guiding principle. This relates to the fundamental objective of developing dementia-friendly and inclusive community initiatives by various municipalities in the four countries and beyond through dementia education, appropriate social programmes, service options and built environmental interventions. The cross-country comparison elucidates that besides environmental factors, public sector areas, for example, healthcare services, and access to public transport, including concession travel passes, could be important contextual factors that influence the level of out-of-home participation among people living with dementia.
